# Architects and Sanatoria

**Published:** 1905-06-24

**Authors:** 


### The Hospital.

A Journal of

The Medical Sciences and Hospital Administration.

Vol. XXXVIII.—No. 978. SATURDAY, JUNE 24, 1905.

### Architects and Sanatoria.

The discussion on Mr. Hall's paper, "Sanatoria for Consumption," reveals the need for a practical architect endowed with great public spirit who will meet the requirements of this country in regard to many hospital buildings in the present day. The steady, continuous, and, we fear, deliberate increase in the cost of hospital and sanatorium buildings has at the bottom, no doubt, a business basis. Cheap buildings mean relatively small fees; costly buildings mean relatively large fees, and the architects, as a body, owe it to their profession, to face this problem of the excessive cost of certain types of buildings, and to supply a remedy. We know as a matter of experience, that cheap buildings can be provided, and, as some of the speakers pointed out, such erections of a relatively temporary and cheap character are often found the most comfortable and suitable for the treatment of patients suffering from acute disease. Too much has been made of the contention, that the modern minutiæ of sanitary detail insisted on by experts involves enormous cost, for it shows a tendency continuously to increase. After all, apart from design, the character of the structure, if the materials chosen be suitable, has very little to do with the efficiency of the treatment, except, perhaps, that temporary buildings are better adapted to the treatment of tubercular cases than any others.

Put in a nutshell, the force of the objections to excessive cost will be seen when we state that *each 1,000 beds*, if provided by local authorities at the present rate of expenditure on some sanatoria in this country, *must cost for buildings alone at least half a million sterling.* Dr. Heron produced some figures in the course of the discussion at the Sanitary Institute on Friday, which revealed the hardships and injustices of the present methods of dealing with tuberculosis where the poorer breadwinner is attacked. He estimates that amongst the poorer classes alone there must be 100,000 of these cases arising each year, which require prompt treatment in a building to be provided by the local authorities. Assuming Dr. Heron is approximately correct, and that each such case, if taken at the earliest stage, must have three months' residence in a sanatorium, 25,000 beds would be required by the local authorities to accommodate this one class of cases. It follows that the outlay on buildings alone for the very poor, under the sanatorium system, must therefore amount to twelve and a-half millions sterling, if the accommodation is to be as costly, for instance, as that at Heatherside, which was inspected last Saturday. To state a fact like this is to condemn the present costly expenditure upon this type of buildings, and to show that for practical purposes it is altogether prohibitory. Can anything then be done to prevent the deadlock with which we are threatened in this matter?

In approaching this problem we must bear in mind that modern treatment is necessarily tending more and more in the direction of taking into these sanatoria those cases only which are found to be in the initial stage of tuberculosis, and who are able to attend to themselves. Such cases can be submitted to a necessary discipline, which includes daily employment, a regulated amount of food, great cleanliness, sufficient exercise, and a continuous residence in the open, with the maximum of sunlight and fresh air. All this is compatible in connection with temporary buildings of the simplest kind. In such circumstances, in our view, an expenditure of £500 per bed upon buildings, or of even half that sum, is not justifiable, for it is practically unnecessary and in fact wholly undesirable. At the present time the architects blame the physicians, and the physicians, for the most part, have not really thought out the matter sufficiently to resist that fetish of wasteful expenditure which is euphoniously described as "sanitary detail insisted upon by experts." When they have done this a practical remedy can soon be applied, for then the physicians could arouse the public to the need of such sanatoria for the poor as they may believe to be justifiable and necessary. The public will thus come to be interested in the matter, and will demand that buildings of this type shall be relatively cheap, say some £100 a bed, apart from the administration block. Urged on by the physicians on the one side and the public on the other, the county councils and municipalities, as the local authorities, would then refuse to entrust work of this description to any architect who does not produce plans of buildings which can be erected at small cost.

It is all very well to urge, as some architects do, that, however costly a sanatorium may be, should it be found, as it may be found within a very few years, that medical science has so advanced as to dispense with this aid to the treatment of tuberculosis, the buildings can be put to some other use. In effect, such an argument should negative the expenditure of the present large sums on any such buildings, for it is a practical certainty, that, should they not be required in the near future for the purposes for which they were built, the initial expenditure will be largely, if not entirely, wasted, whilst the buildings themselves may become a white elephant to their possessors. Common sense, public necessity, the needs of the poorer citizens, and probably the greater facilities for effective treatment in sanatoria, alike demand a reconsideration of the present style of building, and the substitution of something infinitely cheaper and more practicable, or in the alternative the discontinuance of such buildings for the present altogether.

